# Social media and academic success: Impacts of using telegram on foreign language motivation, foreign language anxiety, and attitude toward learning among EFL learners

**DOI:** 10.3389/fpsyg.2022.996577

**Published:** 2022-09-15

**Authors:** Zhongzheng Zhao, Xiaochuan Wang, Sayed M. Ismail, Md. Kamrul Hasan, Arash Hashemifardnia

**Affiliations:** ^1^School of Public Affairs and Administration, University of Electronic Science and Technology of China, Sichuan, China; ^2^Department of English Language, Prince Sattam Bin Abdulaziz University, Al-Kharj, Saudi Arabia; ^3^English Language Institute, School of Humanities and Social Sciences, United International University, Dhaka, Bangladesh; ^4^Department of English, Islamic Azad University of Shahrekord, Shahrekord, Iran

**Keywords:** social media, Telegram application, foreign language anxiety, foreign language motivation, attitude

## Abstract

Concerning the ubiquity of social media, this research tried to examine the impacts of using Telegram on Iranian EFL learners’ foreign language motivation, foreign language anxiety, and attitude toward learning. To achieve these purposes, 60 Iranian EFL learners at the intermediate level were selected and randomly divided into two groups: experimental and control. After that, both groups were pretested on motivation and anxiety variables. After pretesting, the participants in the experimental class received treatment *via* using the Telegram application, and the control students were trained traditionally without using any social media. After an 18-session instruction, a post-test of motivation and a post-test of anxiety were given to both groups, and also an attitude questionnaire was distributed among the experimental group to inspect their attitudes toward the Telegram application in language learning. The results of using two one-way ANCOVA tests showed significant differences between the post-tests of the control and experimental groups in favor of the experimental group. The findings demonstrated that using the Telegram application increased the motivation of the experimental group and decreased their level of foreign language anxiety. Also, the results of one sample t-test showed that the participants of the experimental group held positive attitudes toward using the Telegram application in English language learning. The implications of this research can encourage both teachers and learners to use social media-based instruments in English teaching and learning.

## Introduction

The rapid changes in using technology and the frequent use of the Internet have affected the hierarchy of peoples’ needs including instructional needs and communication needs (Trilling and Fadel, 2009; Diamandis and Kotler, 2012). Using technology in the educational system has significantly provided learners with several opportunities to act efficiently at this age (Suwastini et al., 2020; Puspitasari et al., 2021). The existence of technological tools such as online instruction helps the students learn more flexibly and understand that traditional classrooms are not the only place to learn and teach the English language (Dantes et al., 2019; Putra et al., 2020; Puspitasari et al., 2021; Utami et al., 2021).

One of the most common technology-based methods that has gotten too much popularity nowadays is social media. According to Kaplan and Haenlein (2010), social media refer to a group of Internet-based applications that are built on the technological and ideological basics of Web 2.0 and permit the exchange and creation of user-generated content. Social media allows users to make exclusively recognizable profiles animated by both user- and system-supplied information. Also, social media platforms help users to articulate connections that can be traversed and viewed by others. These connections are usually demonstrated in the forms of followers’ lists, friends’ lists, liked pages, group memberships, and so on (Ellison and Boyd, 2013). Zhang and Leung (2015) explained that the capacity to view and traverse other users’ activities and connections is a distinctive characteristic of social media platforms that is almost unknown in the conventional forms of communication. Social media sites help users to use, generate, and interact with the streams of user-made content provided by their connections (Kane et al., 2014). Users make their content by mixing videos, animations, emoticons, images, text, and so on (Dumpit and Fernandez, 2017). As well as sharing their own content, consumers can use and interact with other users’ content, by sharing, liking, and giving comments on them (Smith, 2017; Masrom et al., 2021).

One of the social media types is the Telegram application that offers several opportunities for online learning. Telegram has some great features including an integrated consumers’ address book (Ghorbani and Ebadi, 2020), stickers for expressing conversations (Banafshi et al., 2020), and group channels for sharing information (Alizadeh, 2018). Recently, investigations have discovered that Telegram positively contributes to creating a stress-free and enjoyable learning setting for increasing learners’ engagement, motivation, and curiosity, and decreasing their level of anxiety (Habibi, 2018; Banafshi et al., 2020; Rostami and Khodabandeh, 2020; Vahdat and Mazareian, 2020). In addition, the Telegram application offers interactive learning activities and extensive material presentations (Manik and Suwastini, 2020; Momami, 2020; Namaziandost et al., 2021).

The Telegram application transfers data and various means of social media to a Messenger network with more than 5,000 users. It manages all kinds of documents that can be accessed by smart mobiles and computers. Telegram, like a mobile phone, can write and read an email list (Ghobadi and Taki, 2018; Namaziandost and Çakmak, 2020), additionally, it can be downloaded freely by students to be implemented in learning and teaching. Based on Sarvari and Ezzati (2019), Telegram bridges the gaps between the students and the teachers when the students are absent from attending real classes. To check students’ advancement, the instructors use the Telegram social media platform. This application makes language teaching and learning accessible to all and increases the motivation of the students to learn language virtually.

Motivation as a kind of motivational state has an influential role in English language learning. Motivation is generally viewed as a student’s desire and inclination to be involved in or make efforts to perform some tasks (Zhou, 2012; Vadivel et al., 2021). Without having the desire to learn, learners are less likely to cooperate, take self-responsibility, or be completely involved in the process of language learning. Dörnyei (2001) states that motivation is considered a vital emotional state that influences second language learning achievements. In addition, Gredler et al. (2004) assert that motivation is the trait that pushes people to conduct some activities. Csizér and Dörnyei (2005) also state that motivation is a construct that describes why a human behaves as he/she does rather than how successful his/her behaviors will be. Dornyei and Ushioda (2011) view motivation as an element of enjoyment. Furthermore, they mention that motivation pushes people to decide, to involve in action, and to try and carry out the activities. A student is motivated when he/she tries to determinedly achieve goals and does her/his best to get them by applying strategies and approaches (Lovato, 2011; Boo et al., 2015; Liu et al., 2021). Several investigations confirmed the positive effects of having much motivation on developing the students’ academic performances (e.g., Arabmofrad et al., 2019; Karabatak and Polat, 2020; Mammadov et al., 2021).

The other emotional state that can be influenced by social media is foreign language anxiety, which is defined as situation-specific, trait, and state (MacIntyre, 2007). Trait anxiety refers to people’s predispositions to get anxious; state anxiety is ephemeral and relates to apprehensions that we experience at a specific moment, and situation-specific anxiety refers to experiencing anxiety in specific situations. Nevertheless, these three sorts of anxiety may not essentially be independent aspects, as revealed by recent studies that discovered that situation-specific and trait anxieties can be correlated to each other (Dewaele, 2013; Galante, 2018). Having anxiety causes EFL students who are not yet skillful to conduct poorly in learning the English language. Since anxiety is an affective factor that can be preventive for students in enhancing language learning (Dornyei, 2005), examining EFL education that can reduce anxiety is paramount.

Using social media as an innovative teaching method can change the attitudes of EFL learners toward language learning. According to Richards and Schmidt (2002), attitude is the opinions and feelings that users of diverse languages or language varieties show toward each other’s languages or their own language. Expressions of negative or positive feelings toward a language may mirror impressions of linguistic difficulties or simplicities, easiness or difficulties of learning, the extent of significance, elegance, social position, etc. Students’ attitudes can be described as a collection of feelings concerning language use and its position in the community (Ahmed, 2015). Attitudes toward a language may also present what individuals feel about the users of that language. Learners’ attitudes play a crucial role in maximizing teaching and learning outputs.

Based on the importance of the variables explained above, this research intended to investigate the effects of applying social media (Telegram application) as the independent variable on Iranian EFL learners’ foreign language motivation, foreign language anxiety, and attitude toward learning dependent variables.

## Review of the literature

The digital era and its modern technologies have substantially altered the ways of communication among people (Yadav et al., 2018). Today, technology, with its non-stop advances, has made some changes in learning and teaching (Hollands and Escueta, 2019). It has provided many opportunities for EFL students to connect with native speakers in an easy way (Reinders and Benson, 2017). The incorporation of technologies in the instructional contexts has brought auspicious chances for teachers and students to rise the effectiveness of the pedagogical processes (Yenkimaleki and van Heuven, 2019). In this respect, Spector and Yuen (2016) described instructional technologies as a theory and act of designing, making, utilizing, and assessing learning resources and processes.

Zengin and Aksu (2017) asserted that instruction has been influenced by technological improvements, including the internet, emails, computers, digital games, and mobile devices and programs. Boo et al. (2015) mentioned that applying technologies such as computers has a crucial role in the multisensory collection of texts, sounds, pictures, videos, animations, and hypermedia to supply meaningful situations to accelerate language learning. Therefore, it can be stated that it is commonly accepted that digital instruments are useful in learning attainment.

One of the most common technologies that are used frequently in learning and teaching is social media, which is applied by 49% of people across the globe (Tankovska, 2021). Social media exists in the form of blogging applications, micro-blogging applications, audio sharing applications, social networking sites, academic networking services, voice over the internet, and others (Chisenga et al., 2014). Regarding the positive effects of social media on learners’ routine life, numerous specialists and researchers have discovered the positive uses of social media for learning and teaching, including language learning (Lau, 2018; Wu and Marek, 2018; Hamadeh et al., 2020) and English language learning (Ismail et al., 2019; Listiani et al., 2021). The majority of them verified that using social media appropriately can generate positive effects on both language learning and teaching.

The Telegram application is a type of social media that belongs to the Social Networking Service (Alizadeh, 2018). This application is a cloud-based social media application, implying that it can easily move conversations between smartphones, the web, and desktops. The Telegram application can share videos and pictures, transfer documents, and send current locations easily. Telegram has different features like those used as social media. The primary element of the Telegram application is almost the same as most other social media applications that can be utilized for conversation communication (chat), there is group chat, and sending files without size limitations. It is better than other social media as it does not have size restrictions for sending files (Aisyah et al., 2021).

Some theoretical perspectives support social medial in language teaching and learning. Two of them are the collaborative learning argument and sociocultural theory. The first perspective believes that social networking platforms can be used to make collaborative learning easier and inspire pupils to more beneficial learning engagements (Lampe et al., 2015; Eid and Al-Jabri, 2016; Raza et al., 2020; Hoi, 2021). Based on this perspective, the social and interactive characteristics of social networking platforms can be used to share information, conduct group work, receive feedback, and make interactions with teachers easier (Chawinga, 2017; Smith, 2017; Al-Rahmi et al., 2020; Ansari and Khan, 2020). Social networking platforms emphasize cooperation and group involvement as opposed to individual learning, thereby permitting learners to get active partners and socially involved in the process of sharing materials, discovering knowledge, and removing problems, which must develop their general learning and academic performances (Lampe et al., 2015; Sarwar et al., 2019; Astatke et al., 2021).

Based on the second perspective, knowledge is created cooperatively within social contexts. It views learning as a condition wherein learners generate their personal meanings from the materials and content delivered to them, rather than trying to memorize the information (Vygotsky, 1978). Additionally, the sociocultural theory is based on the notion that learning can be developed and constructed to be more useful within the orbits of social processes in cognition groups. Furthermore, knowledge is a constant process that needs development with time, and learning occurs better when it follows social perspectives in constructive and effective processes (Brown et al., 1989). Based on Bhattacharjee (2015), the development of constructivism research in the recent age has developed the instruments and focus of media technologies for the quick transformation of knowledge and information to the next generations. Likewise, as offered by Stabile and Ershler (2015), learning is a procedure that leads to the transformation of culture, which may appeal to constructivists to re-examine the effects of social media on culture. The recent advent of social media has enormously influenced attitudes toward instruction by altering the landscape of information accessibility.

In sociocultural theory, learning and teaching need to concentrate on using content to improve tools of understanding, and these contents have got plentiful and simply accessible *via* social media. The impacts of social media, according to sociocultural theory, include important modifications to the ways learners usually communicate, and how they learn basic skills. Therefore, as social media allow the integration, distribution, and alteration of information, it has immense effects on the learning of the students. The benefits of social media platforms follow the rules backed by the constructivism theory (Kelm, 2011). For example, Churcher (2014) indicated that social media platforms result in online communications and sharing of information. Other research indicated that social media tools can facilitate social interactions, communication, participation, the use of current technologies, collaboration, the use of online programs, and the construction of personal meanings that satisfy the learning situation of constructivism (McLoughlin and Lee, 2010). Likewise, the sociocultural theory holds that information, personal activities, and social interactions can be collected by modern instruments of technology (Golub, 1988).

To check the effectiveness of using social media programs, several studies were examined. Chotipaktanasook (2016) examined the impacts of social media platforms on the willingness to communicate among EFL learners. The findings showed applying social media rose the willingness of the students to communicate more. Mompean and Fouz-González (2016) inspected the effectiveness of Twitter as a social media for developing pronunciation learning. Their objective was to find out if social media could boost online engagement and if it might assist learners to pronounce difficult words better. The results indicated that using Twitter as a kind of social media generated positive effects on pronunciation learning.

Zarei et al. (2017) aimed at examining the influences of Telegram on Iranian EFL students’ vocabulary development and their attitudes toward vocabulary learning. To reach these goals, a panel of 100 Iranian EFL students with advanced levels took part in this research. They were chosen after they took the Oxford Quick Placement Test and they were assigned to control and experimental groups. The vocabulary instruction for each group lasted three weeks. The instruction was similar for the two groups except for exercises performed after the class. The experimental participants were required to fill out an attitude questionnaire after the instruction. Then, a vocabulary post-test was given to the subjects of the two groups. The outcomes of the study demonstrated that the experimental participants outdid the control participants on the post-test. The results of the attitude questionnaire revealed that the students presented positive attitudes toward applying the Telegram application.

Tabrizi and Onvani (2017) investigated the effects of Telegram as a kind of social site on vocabulary learning of Iranian EFL students. To attain this goal, 31 Iranian earners, aged 10–14, were chosen using the convenience sampling method. The researcher instructed the English vocabulary to students using two different methods: for one month by applying Telegram and for another month *via* the conventional face-to-face classroom instructions. The results of their study confirmed that using Telegram was more effective than using the conventional methods of vocabulary learning.

Shirinbakhsh and Saeidi (2018) investigated the effects of social media, more particularly Telegram, as an innovative method for training reading strategies in IELTS preparatory courses. The participants of the research were ILETS students studying IELTS preparatory courses in Isfahan, Iran. The participants were randomly divided into an experimental group and a control group, each including eight students. The control group was trained in the strategies by the conventional method, while the experimental group was taught the strategies by Telegram. The results of their investigation depicted that the experimental participants outflanked the control group in the post-test. The findings indicated that the majority of the students confirmed that they prefer to learn by Telegram.

Abu-Ayfah (2019) inspected the EFL College learners’ perspectives on applying Telegram for English language learning. The respondents of the research were 300 EFL college learners, 200 female students, and 100 male students chosen at random from Tibah University in AL-Medina AL-Manwarah in Saudi Arabia. This research followed a quantitative method in which a survey questionnaire was applied as a tool for the collection of data. The findings showed that most EFL learners considered Telegram as a beneficial instrument for learning the English language, especially learning vocabulary.

Alodwan (2021) investigated the effects of employing the Telegram application on enhancing the writing skill of EFL learners. The subjects of the research were 75 who were a two-year Bachelor of English at The World Islamic Sciences and Education University in Jordan. The participants were assigned to two groups: control and experimental. The experimental group was trained by Telegram but the second group was trained traditionally. The results of the research indicated that there were noticeable differences between the means of the experimental participants and the control participants in the writing skill post-tests due to using the social networking Telegram in favor of the experimental participants.

Ammade and Khatimah (2021) examined the effects of the Telegram application on the English reading of the students. The data of this research were collected using a reading test to know the impacts of Telegram on students’ reading ability, and A questionnaire was administered to get data on Telegram’s efficacy on students’ learning of English reading. The findings showed that the Telegram application could support the students’ learning of English reading. The results of the questionnaire indicated that the students agreed that using the Telegram application was effective in English reading.

In another research, Mohd Dollah et al. (2021) examined the effectiveness of the Telegram application on developing ESL students’ writing proficiency. A quasi-experimental design including pre-test and post-test was used in this research. The outcomes indicated that the utilization of the Telegram application improved ESL students’ writing skills.

Meilia Rasiban (2021) investigated the effects of the Telegram application on Japanese language learning as a course for beginner Japanese students. The instruction concentrated on the Telegram application for primary Japanese language learners with various professional backgrounds. The findings indicated a remarkably significant relationship between rising motivation and interest in learning on learning outcomes and Japanese language abilities. The learners were more involved in Japanese language learning and more motivated to learn Japanese after the instruction. The researcher concluded that using the Telegram program was extremely effective in learning the Japanese language.

Having conducted a meticulous literature review, we found that social media has become widespread in all aspects of today’s life, especially in our educational systems. The explanations and empirical studies show that using different social media platforms can generate positive and constructive effects on English language learning. Most conducted studies examined the effects of social media on language skills and language emotional states were ignored. Consequently, this research raised the following research questions to cover this gap.

**Research Question 1.** Does the Telegram application as a sort of social media platform generate positive impacts on EFL learners’ foreign language anxiety?

**Research Question 2.** Does the Telegram application as a sort of social media platform generate positive impacts on EFL learners’ foreign language motivation?

**Research Question 3.** Does the Telegram application as a sort of social media platform help EFL learners to present positive attitudes toward English language learning?

## Methodology

### Participants

The participants of the present research were 60 Iranian EFL learners who were selected among 87 students. They have studied English as a foreign language for 5 years at one of the English institutions in Ahvaz, Iran. The participants’ general English was considered intermediate on the basis of the findings of the Preliminary English Test (PET). The subjects of our study were chosen based on the convenience sampling method. Due to the gender separation in Iran, we could include only males in our research. The selected subjects were assigned to two groups of experimental and control at random.

### Instruments

For homogenizing the general English proficiency of the respondents, a version of the PET test was given to them. Due to some restrictions, only the sections of reading, grammar, and vocabulary of the test were utilized in this investigation. We piloted the test on other similar participants and allocated 55 min for answering all its items. Its validity was acknowledged by three English specialists and its reliability was estimated at 0.89.

The other instrument was an attitude questionnaire created by the researchers themselves. Based on Dörnyei and Taguchi (2010), the questionnaire is one of the most common techniques for gathering research data. We employed a questionnaire to gather data on the participants’ viewpoints regarding their attitudes toward using the Telegram application in learning the English language. There were 30 items in this questionnaire and each item had five options that the students were required to carefully tick off them. The Likert-type items were used in this instrument to show the extent of disagreement and agreement from 1 to 5, which were: completely disagree, disagree, no idea, agree, and completely agree. The questionnaire items were prepared with exact care to guarantee the quality, clarity, standard, appropriateness, reliability, and validity of the questionnaire. Three English professors with teaching experiences of more than 17 years verified the validity of the instrument. The reliability index of the questionnaire was estimated at 0.87 using Cronbach’s alpha formula.

This research utilized a Persian version of the Foreign Language Classroom Anxiety Scale (FLCAS) created by the researchers themselves. All items of the FLCAS were translated into Persian to avoid any problems that Iranian EFL learners could face in answering the items. Then three English professors who had gotten their PhD in TEFL examined the wordings and content of the Persian version of this scale and verified its validity. After that, Cronbach’s alpha formula was run to calculate the reliability of this tool and the results showed that the reliability index was 0.81. Thirty-three items in the form of a five-point Likert scale were included in this questionnaire and they were used as the anxiety pre-test and post-test of this research.

The last research instrument was a questionnaire extracted from Gardner (2004) Attitude/Motivation Test Battery (AMTB). The original version of the questionnaire had 104 items but we selected those 74 items that measured the instrumental and integrative motivation, and attitude toward language learning. The items were in the form of a 5-point Likert-type from completely disagree to completely agree. All items were translated into Persian to help the participants understand them well. The validity of the AMTB was verified by those who examined the validity of the FLCAS. The reliability index of this scale was 0.84 based on the statistical findings of Cronbach’s alpha. We used the AMTB questionnaire both as the motivation pre-test and post-test of the present research.

### Procedures

To perform this study, first, 60 Iranian EFL students were chosen and divided into two groups: control and experimental. Next, both groups were pretested on motivation and anxiety. After that, the participants in the experimental group received treatment *via* the Telegram application, and the control students were trained traditionally without using any social media. Six lessons from the Top Notch 2 book were trained to the experimental group using Telegram. Each lesson was trained in two online sessions. The materials and content were sent to the experimental group at a certain time and the researcher explained them to the participants in the Telegram application. The participants were requested to practice and learn the materials cooperatively and asked the teacher only those hard parts that were incomprehensible to them. The teacher provided constructive feedback whenever the students asked for help. It should be noted that the conversation, vocabulary, and grammar of each lesson were instructed to the groups. The same materials were traditionally trained to the control group. The researcher personally attended the class and tough the control group in a face-to-face situation.

The instruction took 18 sessions, each took 55 min. The participants’ homogeneity was guaranteed in the first session; they were pretested on motivation and anxiety in the second and third sessions; in the 12th session, the groups received the treatment. In the 16th and 17th sessions, the motivation and anxiety post-tests were administered, respectively. In the last session, the attitudinal questionnaire was distributed among the experimental participants to check their attitudes toward the use of the Telegram application in language learning.

### Data analysis

Having gathered the data, we analyzed them based on the research questions. Therefore, we used one-way ANCOVA to see if the differences between the post-tests of the control and experimental groups were significant or not. Also, we used one sample t-test for analyzing the data of the attitudinal questionnaire to understand if they presented positive attitudes or not.

## Results

To analyze the gathered data, we applied the SPSS software and reported the outcomes in Table 1.

**TABLE 1:** Descriptive statistics of both groups on the anxiety post-tests.

Groups	Means	Std. deviations	N
Control	60.10	10.44	30
Experimental	104.36	19.88	30
Total	82.23	27.31	60

As seen in Table 1, the control group’s mean score is 60.10 and the experimental group’s mean score is 104.36. It appears that the experimental participants got better scores than the control participants on the anxiety post-test. To find out the difference between the anxiety post-tests of both groups, a one-way ANCOVA test was used in Table 2.

**TABLE 2:** Inferential statistics of both groups on the anxiety post-tests.

Source	Type III sum of squares	df	Mean squares	F	Sig.
Corrected Models	34663.47^a^	2	17331.73	105.46	0.00
Intercept	791.48	1	791.48	4.81	0.03
Pre	5270.41	1	5270.41	32.07	0.00
Group	28662.99	1	28662.99	174.41	0.00
Error	9367.25	57	164.33		
Total	449770.00	60			
Corrected Total	44030.73	59			

^a^*R*^2^ = 0.787 (Adjusted *R*^2^ = 0.780)

According to the results indicated in Table 2, the variations between the anxiety post-tests of the experimental and control participants were statistically significant. Based on the significant values presented in Table 2, we can conclude that the experimental participants outdid the control participants on the anxiety post-tests. The treatment could help EFL learners reduce their anxiety levels during learning English.

In Table 3, the descriptive statistics of the two groups on the motivation post-tests are displayed. The experimental participants’ mean score is 131.86 and the participants’ mean score is 119.66. According to the mean scores, we can say that the experimental students outdid the control students on the motivation post-tests. This claim can be rejected or accepted by conducting a one-way ANCOVA test, as shown in Table 4.

**TABLE 3:** Descriptive statistics of both groups on the motivation post-tests.

Groups	Means	Std. deviations	*N*
Control	119.86	20.95	30
Experimental	131.66	25.95	30
Total	125.76	24.12	60

**TABLE 4:** Inferential statistics of both groups on the motivation post-tests.

Source	Type III sum of squares	df	Mean squares	F	Sig.
Corrected Models	15682.51^a^	2	7841.25	23.93	0.00
Intercept	1390.74	1	1390.74	4.24	0.04
Pre	13593.91	1	13593.91	41.50	0.00
Group	1594.34	1	1594.34	4.86	0.03
Error	18670.21	57	327.54		
Total	983388.00	60			
Corrected Total	34352.73	59			

^a^*R*^2^ = 0.457 (Adjusted *R*^2^ = 0.437).

The results in Table 4 show that the *Sig* value (0.00) is less than 0.05, this implies that there exists a meaningful difference between the motivation post-tests of both groups. Indeed, the experimental participants outflanked the control participants on the motivation post-tests. This betterment can be ascribed to social media (Telegram application). Using the Telegram application generated positive effects on students’ language learning that resulted in increasing their learning motivation.

In the questionnaire shown in Table 5, all the mean scores of the questionnaire items were well above 3.00 (which is the average value of the choices where completely agree receives 5.00 and completely disagree receives 1.00). This implies that the experimental group of students agreed with all the questionnaire items, which were all positive comments about Telegram and applying it to learning English. The highest mean scores out there belonged to items # 6 and 30 (*M* = 4.73 and 4.80, respectively) through which the learners expressed that (a) learning through social media motivated them to succeed, and (b) using social media for learning purposes develops their academic performances. The overall mean score of the 30 items of the questionnaire equaled 4.34, as is also depicted in Table 6.

**TABLE 5:** Participants’ attitudes toward using social media.

	Completely disagree	Disagree	No idea	Agree	Completely agree	Mean
(1). Using Telegram gives me more room to express myself.	0	0	10	1	19	4.60
(2). I make my course assignments and projects applying social media applications	2	3	5	13	7	3.63
(3). Social media motivate students to learn more than the traditional methods.	0	1	3	16	10	4.16
(4). Using Telegram contributes to my personal development.	5	0	5	13	7	3.56
(5). Social media rises willingness to communicate among students.	0	0	3	17	10	4.23
(6). This Telegram motivates me to succeed.	0	0	0	8	22	4.73
(7). I would like to use Telegram when I become a teacher.	0	0	2	16	12	4.33
(8). I think Telegram makes learning easy.	0	0	5	11	14	4.30
(9). Using social media rises students’ confidence toward EFL learning.	0	1	5	12	12	4.16
(10). I like to attend classes where the instructors use social media in their teaching.	0	0	1	12	17	4.53
(11). Communicating with my teachers and classmates utilizing social media provides me with a good learning experience.	3	0	2	10	15	4.13
(12). Social media inspire me to learn better than the conventional teaching approaches.	0	0	3	10	17	4.46
(13). Using social media for learning and teaching seems desirable.	0	0	1	10	19	4.60
(14). Social media positively affects my attention span	0	2	5	11	12	4.10
(15). I am pleased with applying social media for my learning.	0	0	1	11	18	4.56
(16). Using social media develops self-study.	1	3	5	7	14	4.00
(17). Telegram can be used at any time.	2	2	3	12	11	3.93
(18). Social media are useful for teaching all skills.	0	0	0	12	18	4.60
(19). Using social media is less boring than the traditional method.	0	0	0	10	20	4.66
(20). Using social media is good for shy students.	0	0	2	12	16	4.46
(21). Social media such as Telegram improves my communication with teachers and classmate.	0	1	2	17	10	4.20
(22). Social media such as Telegram helps me become an independent learner	0	0	0	19	11	4.36
(23). Social media such as Telegram reduces learners’ anxiety toward EFL learning.	0	0	2	13	15	4.43
(24). Social media rises positive attitudes toward EFL learning	0	0	0	9	21	4.70
(25). Debating ideas and sharing opinions with others through social media develops my critical thinking abilities.	0	1	3	12	14	4.30
(26). Learning via social media develops self-independent learning for me.	0	2	2	14	12	4.20
(27). I express my ideas and thoughts more freely with social media than in face-to-face discussions with my teachers and friends in the classrooms.	0	0	0	10	20	4.66
(28). Interacting with the class group on social media aids me to develop my social capabilities.	0	0	0	13	17	4.56
(29). Social media assist me to learn cooperatively with those who have similar interests.	0	0	3	11	16	4.43
(30). Applying social media for learning purposes develops my academic performance.	0	0	0	6	24	4.80

**TABLE 6:** Descriptive statistics for experimental participants’ attitudes scores.

	*N*	Means	Std. deviations	Std. error means
Attitude	30	4.34	0.30	0.05

The overall mean score of the questionnaire was well above 3.00, which showed that the participants had a positive attitude toward the instruction they had received *via* Telegram. To figure out if the positive attitudes reached statistical significance, a one-sample *t*-test was run.

It is clearly observed in Table 7 that the participants’ attitude was significantly positive as the *p-*value was smaller than the significance level (*p* < 0.05). The participants of the experimental group, thus, did welcome using Telegram for learning the English language in Iranian EFL contexts.

**TABLE 7:** One-Sample *t*-test results for the experimental participants’ attitudes scores.

	Test value = 0					
	T	df	Sig. (2-tailed)	Mean difference	95% Confidence interval of the difference	
					Lower	Upper
Attitude	78.07	29	0.00	4.34	4.23	4.45

## Discussion and conclusion

After gaining the results, it was shown that the experimental participants had better performances than the control participants both on the anxiety and motivation post-tests. It was discovered that using social media (Telegram application) reduced the participants’ anxiety levels and increased their learning motivation. In addition, it was revealed that Iranian EFL students presented positive attitudes toward applying the Telegram application in learning English.

The outcomes of our research are in accordance with the previous research confirming the positive impacts of using social media on developing English language learning. For example, our research results are in agreement with Zarei et al. (2017), who investigated the impacts of Telegram on the developing vocabulary knowledge of Iranian EFL students. Their results demonstrated that using Telegram generated positive effects on the experimental group participants’ vocabulary knowledge.

Besides, the findings of this research are in agreement with Tabrizi and Onvani (2017), who confirmed the effectiveness of using Telegram as a kind of social network for learning L2 vocabulary by Iranian EFL learners. The other study that confirms our results is the study that was conducted by Shirinbakhsh and Saeidi (2018), showing that applying Telegram was an effective tool for training reading strategies in IELTS preparatory courses.

Additionally, our outcomes are in accordance with Alodwan (2021), who checked the impacts of the Telegram application on developing writing skills and concluded that using Telegram as a kind of social networking developed the writing skill of the students in the experimental class. Furthermore, our findings are computable with Meilia Rasiban (2021), who inspected the effects of the Telegram application on Japanese language learning. His results depicted that using the Telegram application assisted the students to be more involved in Japanese language learning and become more motivated to learn Japanese after the instruction.

Moreover, the outcomes of this research are in line with Barton et al. (2018), who verified the positive impacts of using social media on students’ motivation, attention, and academic performances. The findings of other studies carried out by Azabdaftari and Mozaheb (2012), Amemiya et al. (2007), Tozcu and Coady (2004)Chen et al. (2008), and Basoglu and Akdemir (2010) are similar to our findings.

Concerning the positive attitudes of Iranian EFL students toward using the Telegram application, our results are in line with Abu-Ayfah (2019), who surveyed the perceptions of EFL college students about the uses of Telegram in English language learning. His results revealed that the students presented a positive attitude toward the Telegram application and most of them perceived Telegram as a useful device for English language learning. Also, our findings confirm those findings gained in the research done by Moulishree et al. (2020), which showed that the participants of their study held favorable opinions about using social media in language learning.

In addition, our outcomes are the same as Zarei et al. (2017), whose research participants presented a positive attitude toward applying the Telegram application in English language learning. Besides, the outcomes of our research are in line with Sharma (2019), who showed that Saudi EFL learners perceived social media as a beneficial strategy to develop English language learning. Additionally, this part of our outcome is in line with Shirinbakhsh and Saeidi (2018), who indicated that the participants in their study preferred using Telegram other than using traditional methods in learning reading skills.

Our results are supported by the collaborative learning theory, which states that utilizing social media can facilitate collaborative learning and encourage learners to be more engaged in the learning process (Eid and Al-Jabri, 2016; Raza et al., 2020). Based on this theory, social networking sites increase the interactions between the students and teachers and help teachers to provide more feedback to the students. In addition, our findings are advocated by the sociocultural theory, which believes that knowledge is generated in a collaborative way within social contexts. Based on sociocultural theory, social media allow the integration, distribution, and alteration of information among students.

One reason behind our results can be the opportunity that the Telegram application provided for the students and teachers to create fun, private, simple, friendly, interesting, and comprehensive relationships both inside and outside of the class. The other reason can be ascribed to the nature of Telegram, which is interactive and provides discussions and constructive feedback for the learners to boost their English language learning optimally. Telegram as a useful platform has some effective properties including the ease of sharing personal opinions and peer inputs and inputs from the teachers to individual students. These features can assist EFL students to learn English more simply.

The other explanation for our results can be attributed to the ubiquity of Telegram, which contributes to exchanging personal ideas and peer feedback as well as the teachers’ feedback to individual students. The other possible justification for our findings is that the participants of the experimental group did not attend the real class to face the teacher. Lack of facing with the teacher can reduce the anxiety of the students and also can increase the motivation of the shy and introverted students. One more justification for the obtained findings can be the fact that using Telegram permits the learners to learn and practice the English language at any time and any place they want. There is no time and place limitations while using social media for language learning; therefore, the learners can learn the materials at the place and time that they like the best.

Our findings can bring about constructive implications for EFL learners and teachers. The findings can encourage students to use different sorts of social media sites and instruments while trying to learn a new language. By using social media, the students can learn by themselves and develop their self-study and autonomy. In addition, EFL learners can utilize the Telegram application to enhance their English language outside the classroom. Using the Telegram application can stimulate self-study and change the role of the learners. By using social media, EFL learners can access the world’s information easily and quickly. Therefore, they can understand that the world is in their hands and they can access a wealth of information that can rise their language learning motivation. In addition, using the Telegram application allows students to communicate *via* text and voice messages. Voice messaging is a relatively new feature that aids the students to develop their speaking and pronunciation skills by recording their answers in the chats. The Telegram application uses cloud storage so students can access teachers’ conversations on different platforms, as mentioned earlier.

The results of this research can be beneficial for teachers. By using Telegram, the teachers can simply share the materials, and also, they discuss the materials with the learners freely. Besides, both instructors and students can experience a new teaching and learning process through Telegram. Our findings can persuade EFL teachers to utilize social media platforms in their classes to make lovely and lively contexts for learning. The social networks can assist EFL instructors to do their best to blend technologies and recognize barricades to technology incorporation. The spread of social media helps language teachers to develop social learning beyond the classrooms. Social media platforms have changed the conventional teacher-centered approaches and require instructors to be more innovative in adapting and customizing their own materials. In teacher-centered classes, students have passive roles in learning and they cannot take part in classroom activities. The results of this investigation can assist teachers to engage students in the learning process and abandon taking the full responsibility of teaching and bring up independent and autonomous language learners. Educators can also have more student-centered classes using the Telegram application in their teaching.

Shortly, the results of this research confirmed the positive impacts of applying social media (Telegram) on Iranian EFL learners’ foreign language anxiety and foreign language motivation. Also, the findings indicated that EFL students held a positive attitude toward using the Telegram application in English language learning. Based on the findings, it is suggested that teachers use different kinds of social media platforms as complementary tools for face-to-face instruction. In fact, the Telegram application is presently one of the most innovative applications that can be employed in EFL classrooms. One effective way that teachers can aid language learners to learn a foreign language is by utilizing various technologies. One of the technologies that can be simply used to assist students is the Telegram application, which is dominating in most of the learners’ life and is not only a communication application.

Incorporating Telegram channels into existing learning practices can supply informal learning situations and make new opportunities for English learning. Telegram as a social network instrument is getting one of the main devices for entertainment and education. The Telegram application is a social networking application that has numerous stickers with written English expressions and words. Students can even make communications without any texts and only *via* stickers and images; therefore, Telegram has an important effect on communication and learning the English language. Generally, it can be said that due to the efficiency of Telegram as a technological instrument that has already been verified to be welcomed by EFL students in Iran, learning English can also be further facilitated as students can simply associate the meanings of the vocabulary with the interesting stickers on their mobile devices.

We conclude that social media platforms have a vital role in foreign/second language learning. Therefore, it is highly important to encourage learners to utilize technology in their self-learning as well as language teaching because numerous students have much inclination to use technology only for entertainment.

Finally, the limitations of the study were mentioned, and accordingly, some suggestions were recommended. There were only 60 participants in this research; next researchers are required to perform other experimental studies with a large population to increase the generalizability of their outcomes. This research was conducted on males and the females were not included; next researchers are invited to work on the same topic on both genders. The results of this study were collected using quantitative instruments; next researchers are required to use qualitative instruments such as observations and interviews to gain more valid results. Additionally, the next studies are offered to investigate the impacts of different kinds of social media tools and platforms on different kinds of language skills and sub-skills. Next researchers are recommended to examine the attitudes of teachers toward the effectiveness of the Telegram application in their teaching. As this study was conducted on urban students; next studies can work on the same topic considering rural students.

## Data availability statement

The raw data supporting the conclusions of this article will be made available by the authors, without undue reservation.

## Author contributions

All authors listed have made a substantial, direct, and intellectual contribution to the work, and approved it for publication.
